# The Effect of Applying Robot-Assisted Task-Oriented Training Using Human-Robot Collaborative Interaction Force Control Technology on Upper Limb Function in Stroke Patients: Preliminary Findings

**DOI:** 10.1155/2021/9916492

**Published:** 2021-07-28

**Authors:** Qingming Qu, Yingnan Lin, Zhijie He, Jianghong Fu, Fei Zou, Zewu Jiang, Fengxian Guo, Jie Jia

**Affiliations:** ^1^Department of Rehabilitation Medicine, Huashan Hospital, Fudan University, China; ^2^Shanghai Electric GeniKIT Medical Science and Technology Co. Ltd., Shanghai, China; ^3^National Clinical Research Center for Aging and Medicine, Huashan Hospital, Fudan University, China

## Abstract

Stroke is one of the leading causes of death and the primary cause of acquired disability worldwide. Many stroke survivors have difficulty using their upper limbs, which have important functional roles in the performance of daily life activities. Consequently, the independence and quality of life of most stroke patients are reduced. Robot-assisted therapy is an effective intervention for improving the upper limb function of individuals with stroke. Human-robot collaborative interaction force control technology is critical for improving the flexibility and followability of the robot's motion, thereby improving rehabilitation training outcomes. However, there are few reports on the effect of robot-assisted rehabilitative training on upper limb function. We applied this technology using a robot to assist patients with task-oriented training. Posttreatment changes in Fugl-Meyer and modified Barthel index (MBI) scores were assessed to determine whether this technology could improve the upper limb function of stroke patients. One healthy adult and five stroke patients, respectively, participated in functional and clinical experiments. The MBI and Fugl-Meyer scores of the five patients in the clinical experiments showed significant improvements after the intervention. The experimental results indicate that human-robot collaborative interaction force control technology is valuable for improving robots' properties and patients' recovery. This trial was registered in the Chinese clinical trial registry (ChiCTR2000038676).

## 1. Introduction

Stroke is a common cerebrovascular disease that is diagnosed on the basis of clinical features and imaging [1]. Most strokes result from transient ischemic attacks associated with blockages of blood flow [2], while about 10–40% of strokes are attributed to intracerebral hemorrhage [3] caused by the rupture of cerebral arteries. Stroke remains the leading cause of death and disability in China despite substantial advances relating to its prevention and treatment [4]. More than 80% of stroke patients develop acute motor dysfunction, and almost 50% of patients eventually develop long-term motor function limitations [5]. Upper limb (UL) function is essential for executing daily activities. However, persistent UL sensorimotor impairments occur in up to 75% of stroke patients [6] and include paresis, ataxia, spasticity, a reduced range of motion spasticity, and poor spatiotemporal coordination, which significantly affect the quality of life of patients with stroke. Therefore, targeting UL function is a core element of rehabilitation to optimize patient outcomes and reduce disability [7].

Rehabilitation to improve and maintain patients' functions plays a critical role in the recovery process [8]. Many rehabilitative therapies have been applied to improve UL function, such as robot-assisted therapy [9], virtual reality [10], mirror therapy [11], music playing [12], transcranial direct current stimulation [13], motor imagery [14], bilateral motor training [15], task-oriented training (TOT) [16], and constraint-induced movement therapy [17]. Among these approaches, TOT [18] and robot-assisted training are reported to be effective for improving patients' poststroke abilities to execute activities of daily living (ADL) and their UL function [19]. TOT, which targets patient motor function control, entails applying physical training inputs within specific tasks associated with the patient's environment, while providing the patient with appropriate internal and external feedback. TOT can increase muscle strength on the hemiplegic side, correct a flawed compensatory strategy, and help the patient to establish a normal movement pattern [20]. It can simultaneously activate the corresponding expression area of the cerebral cortex and promote remodeling of the central nervous system in the corresponding functional area [21]. Robotic assistance, which enables highly repetitive, task-oriented, intensive, and quantifiable neurorehabilitation treatment to be delivered [22], is considered one of the most promising methods for functional UL restoration. A previous study found that a robot-assisted TOT program could improve the ability of stroke patients to grasp objects [23]. Though several studies have demonstrated the effectiveness of robot-assisted treatment, few studies have examined the application of human-robot collaborative interaction force control technology (HRCIFCT). This technology is critical for improving movement compliance, flexibility, and the followability of the robot's movements, which significantly enhances the effects of the inactive and assisted control modes of rehabilitative training.

We designed a robot-assisted TOT program centering on virtual reality games and entailing different levels of difficulty to improve the effectiveness of rehabilitative training. HRCIFCT was combined with robotic assistance, given its ability to improve the robot's properties, thus offering patients better service. This technology can solve the problem of dynamic compensation and enhance movement flexibility, while also accelerating the starting ability of the rehabilitation manipulator. Importantly, it can judge the patient's intended direction of movement, providing flexible tracking. All of these advantages contribute to making robot-assisted training more effective.

## 2. Materials and Methods

### 2.1. Type of Motion Training

In general, the type of motion training entailed in robot-assisted rehabilitative exercises varies according to the stage or severity of the disease. Three modes of motion training can be identified according to the auxiliary force provided by the robot: passive, assisted, and active motions. The passive mode of motion training is used to assist stroke patients who are unable to perform any kind of movement. The assisted motion mode is utilized to support stroke patients who can execute some kind of movement. Active movement encompasses the entire process of movement. Although it is fully self-initiated by patients, they are unable to complete the movement in a natural manner [24]. HRCIFCT entails a novel design and can significantly benefit patients who can perform an assisted or active movement. At the same time, TOT requires patients to have the ability to participate actively. Therefore, we focused solely on assisted and active modes of movement.

### 2.2. Rehabilitation System

**Figure 1:**
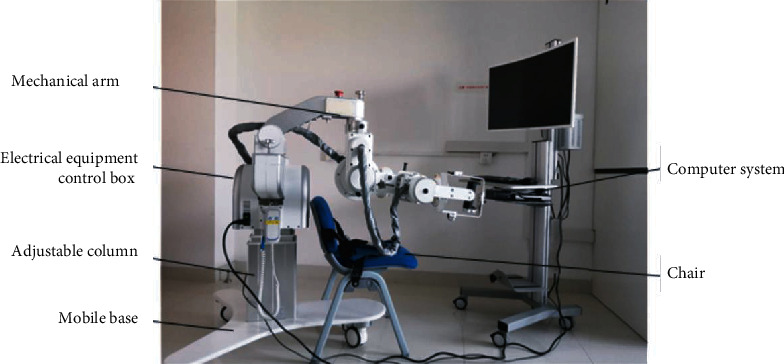
Robot-assisted rehabilitation system.

The rehabilitation system used in this study was the FELXO-Arm1 system manufactured by Shanghai Electric GeniKIT Medical Science and Technology Co., Ltd. and comprises hardware and software components (Figure 1).

FELXO-Arm1 has five degrees of freedom, which is uncommon in rehabilitative therapeutic devices, and is used to help stroke patients recover UL function. It has three passive joints in the horizontal plane and two active joints in the sagittal plane, comprising a motor and gear, which could provide additional assistance to patients undergoing rehabilitative training. The encoder and the torque sensor have different functions. Whereas the former is used for recording angular measurements of joints, the latter is utilized to obtain human-robot interactive torque measurements. Different motion control algorithms can be developed based on the mechanical structure to enable its adaptation for different rehabilitative training modes.

The power unit on the sagittal plane of the joint, which comprises a Maxon EC motor and a Harmonic Drive harmonic gear, complies with the training requirement of robot-assisted UL rehabilitation. Additional 46 Nm and 13 Nm assistive torques can be used for the shoulder and elbow joints, respectively. In addition to the robot, this hardware system includes other components, such as the mechanical manipulator, a 3D force sensor, and a controlling computer.

The software for the motion rehabilitation system was developed by the same company running on the external computer system. To improve instantaneity and operability, the software was designed using a real-time module. It presents a variety of virtual reality games, which can be chosen for specific rehabilitation targets, such as improving the range of motion, cognitive function, and activities of daily living. The parameters of the games include background complexity, running speed, training time, and background music, and the level of difficulty can be set according to the requirements and capacities of different patients.

### 2.3. Human-Robot Collaborative Interaction Force Control Technology (HRCIFCT)

**Figure 2:**
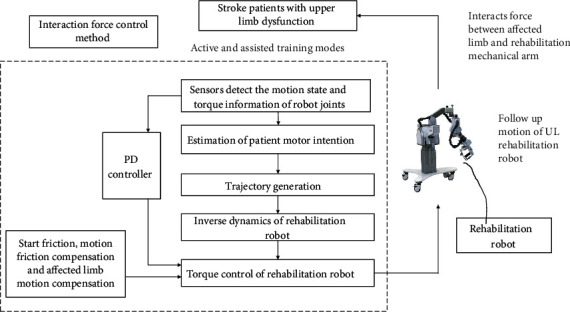
Human-robot collaborative interaction force and interactive control.

Figure 2 presents a schematic diagram of the HRCIFCT, which comprises three parts: a UL rehabilitation robot (ULRR), the patient, and an interactive force control method. The ULRR provides three rehabilitation auxiliary training options, namely, passive, assisted, and active training, and the device is mainly applied in the active and assisted modes. According to the different torque values obtained, we determined that the ULRR could be used to assist patients undergoing active and assisted rehabilitative training. The angle sensor responds to information on the joint's motion in real time, and information on the interaction force between the patient and the ULRR is obtained through the torque sensor installed at the joint position.

**Figure 3:**
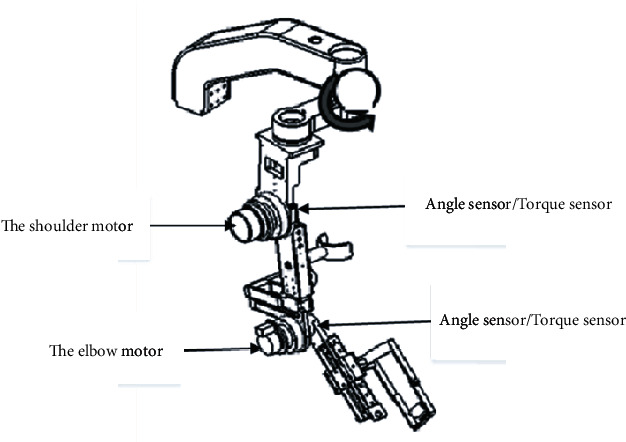
Schematic diagram showing the positioning of the motor and the sensor.

The interactive force control technology enables the patient's movement intention to be estimated. Using this technology, an inverse dynamic model of the ULRR arm, as well as the starting and kinematic friction and the motion compensation of the affected limb, can be established.  Figure 3 presents a simplified model of the UL rehabilitation manipulator, depicting the arrangement of the two joints in the sagittal plane and the points of installation of the motor and sensor. The intended direction of movement can be determined according to the torque value detected by the joint torque sensor.

### 2.4. Task-Oriented Training for Assisted and Active Training Modes

TOT is a theory of rehabilitation characterized by divergence. Clinicians formulate a task to be implemented that is individually tailored to each patient according to their specific functional impairments and training targets. During the training session, the clinicians provide the patients with appropriate feedback, instructing them to maintain good posture and avoid compensation. Several studies have confirmed that a high number of repetitions performed within a single session yield better outcomes [25, 26]. To enhance therapeutic efficacy, the training tasks should be designed to intrigue the patient, thereby ensuring their continuous participation in the training program. Accordingly, we designed training tasks using different virtual reality games to demonstrate the efficacy of applying HRCIFCT to the UL function with robotic assistance.

### 2.5. Selection of Participants

We set the following inclusion criteria for selecting participants. (1) Patients were diagnosed with stroke, as confirmed by imaging and clinical data. (2) The diagnosis was confirmed less than 3 months after a stroke. (3) Patients exhibited mild to moderate motor impairment. (4) Patients exhibited mild spasticity of the affected UL (a modified Ashworth scale score < 2). (5) Patients agreed to participate in robot-assisted rehabilitative training.

The exclusion criteria were as follows: (1) orthopedic injuries of the musculoskeletal system, (2) severe visual or hearing impairments, (3) severe diseases of vital organs (e.g., heart failure or kidney failure), and (4) inability to complete the treatment. We also recruited a healthy volunteer for the functional experiments.

## 3. Experiments and Results

### 3.1. Experimental Scheme

In this study, the functional and clinical experiments were designed to test the effectiveness of the robot-assisted TOT. The training tasks comprised different virtual reality games. A healthy volunteer was asked to perform functional experiments after being instructed on how to use the robot and how to play the virtual reality game. Goals were used to test the practicability of the robot-assisted training system and the volunteer's subjective feelings while completing the task. In addition, five stroke survivors were recruited to participate in the clinical experiments, with the aim of testing the validity and utility of this rehabilitation system. Fugl-Meyer and MBI scores served as the primary measures.

### 3.2. Functional Experiments

**Figure 4:**
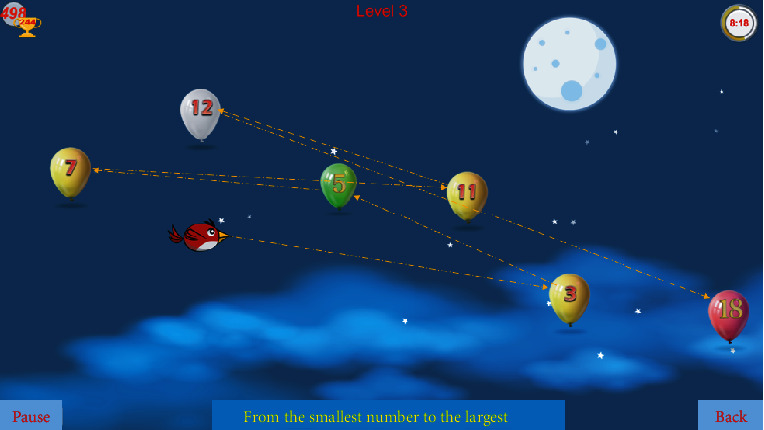
A schematic diagram of game-based task-oriented training.

The objective of the functional experiments was to test the robot-assisted rehabilitation system after incorporating the HRCIFCT to assess whether it elicited subjective feelings of comfort in the subject and provided efficient TOT. After all of the training preparations had been completed and the system had been connected, robot-assisted TOT was performed. One of the activities of daily living (ADL) training games was randomly chosen by a therapist who informed the volunteer about precautions to be taken. Two robot-assisted training modes, namely, assisted and active training, were then tested. Each model was tested continuously for 20 minutes using the same game.  Figure 4 depicts the game content. During and after the training, we asked the volunteer about their subjective feelings. For example, during the assisted training, we asked the volunteer whether they could feel the auxiliary force exerted by the robot, whether this force was appropriate, and other relevant questions. We also asked the volunteer whether the game-based TOT was entertaining and likely to increase their willingness to participate. All of these issues are critical, as they influence patients' comfort and active participation during the training process and have a crucial bearing on patient outcomes. The subject reported a high level of satisfaction during the entire training process and confirmed that the software ran smoothly. Accordingly, we suggest that a robot-assisted rehabilitation system using HRCIFCT is effective in improving the UL function of stroke patients.

### 3.3. Clinical Experiments

The purpose of the clinical experiments was to validate the effect of robot-assisted TOT with HRCIFCT on the UL function of stroke patients during rehabilitation. Five stroke survivors were recruited as participants in the clinical experiments, which were held over a four-week period (more than three times per week for a total of 15 therapy days). Each survivor underwent one session of game-based, robot-assisted TOT that lasted 30 min on one therapy day. The patients also agreed to participate in two-hour daily sessions (5 days a week) that covered other interventions, including physical and occupational therapy according to the degree of their UL impairment and expectations. To evaluate the effect of robot-assisted TOT treatment on the recovery of UL function, the Fugl-Meyer shoulder and elbow coordination (SEC) and MBI scores were assessed. The UL function of the five stroke patients was evaluated before the treatment commenced, after the fifth treatment, and after the last session. Before participating in the experiment, all of the patients underwent a cognitive assessment using Mini-Mental State Examination (MMSE), which showed that it could accurately screen cognitive impairments and was clinically feasible [27].

The Fugl-Meyer assessment, which measures the ability of individuals to move their affected UL, is a well-designed test that has been widely used in the stroke population worldwide and shown to be a valid and reliable measure [28]. The total score for UL function in the Fugl-Meyer assessment is 66. However, we adapted the test to focus on shoulder-elbow coordination and named this revised version Fugl-Meyer SEC. The maximum score for each item in the modified version was 3 points (with a maximum score of 42 points). The MBI is also widely used to assess the ability of individuals to perform daily activities [29]. It comprises 10 items, amounting to a total of 100 points. An evaluation of changes in pre- and posttreatment scores can be indicative of the treatment's effectiveness.

Table 1 presents the baseline demographics and clinical characteristics of the five stroke survivors. The number of days since stroke onset ranged from 16 to 71 days (a mean of 55 days) among the five patients. All five subjects had suffered cerebral infarctions, and the dominant sides of two of the patients had been affected. Three of the patients were men (a mean age of 68.7 years), and two were women (a mean age of 61 years).

**Figure 5:**
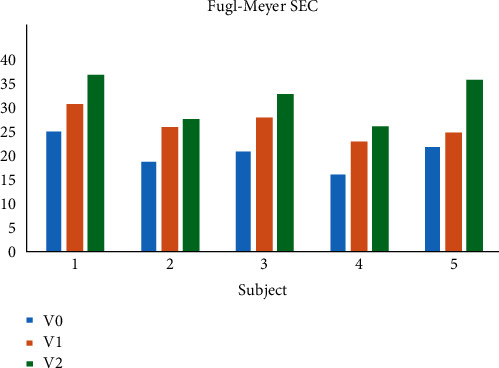
A comparison of the Fugl-Meyer SEC scores of stroke patients.

After undergoing the robot-assisted TOT rehabilitation, all of the participants showed improvement in their shoulder-elbow coordination, as demonstrated by increases in the Fugl-Meyer SEC scores. After the fifth training session, the scores of patients 1, 2, 3, 4, and 5 increased by 5, 7, 7, 7, and 3, respectively. After the fifteenth training session, the scores of the patients increased by 12, 9, 13, 10, and 14 for subjects 1, 2, 3, 4, and 5, respectively. At the conclusion of the experiment, an increase in the score was most apparent for subject 5.  Figure 5, which depicts the Fugl-Meyer SEC scores, reveals an upward trend in Fugl-Meyer SEC scores for the five subjects.

**Figure 6:**
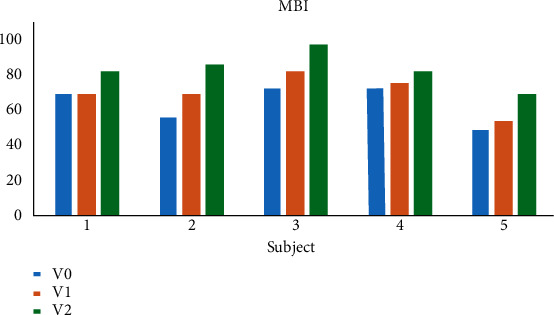
A comparison of the MBI scores of stroke patients. Notes: V0 denotes the initial value, V1 denotes the value after the fifth session, and V2 denotes the value after the last session.

Similar to the Fugl-Meyer SEC scores, the MBI scores for the five patients also increased (Figure 6). Increases in the patients' scores for the second evaluation ranged from 0 to 13, being 0, 13, 9, 3, and 6 for subjects 1, 2, 3, 4, and 5, respectively. For the last assessment, increases in scores ranged from 10 to 30, being 13, 30, 25, 10, and 21 for subjects 1, 2, 3, 4, and 5, respectively. Of the five patients, only subject 1 did not achieve an increased MBI score in the second evaluation. However, in the last evaluation, the MBI score of subject 1 increased by 13 points. The MBI score of subject 4 increased by 10 points after completion of the treatment, which was the lowest increase among the five patients. Subject 2 showed the most dramatic increase, with a total gain of 30 points.

## 4. Discussion

With advancing technology, an increasing number of novel strategies are being developed and applied within clinical rehabilitation, bringing significant improvements to the UL function of stroke patients. Robots used in rehabilitation training are becoming increasingly critical for stroke patients [19] because they can provide a variety of targeted training modes for helping patients to recover lost or impaired functions. Previous studies have highlighted the importance of robot-assisted technology in the recovery of UL function [30]. TOT associated with motor relearning is widely used within rehabilitation programs and is being used to improve UL function [31]. To date, several TOT protocols have been developed to train patients, such as constraint-induced movement therapy, which is a specific TOT that can lead to enhanced motor function of the affected UL [32]. Using robot-assisted training combined with TOT is more efficacious than applying robot training on its own to improve the limb function of stroke patients [33]. Accordingly, we aimed to assess and validate the performance of robots when HRCIFCT was incorporated within a robot-assisted TOT program.

The results for the treatment outcomes of the five patients indicated that both the Fugl-Meyer SEC scores and the MBI scores showed improvements at the conclusion of the training program, although the MBI score of subject 1 showed no change after the second assessment. Moreover, scores obtained using both methods increased above the minimal clinically important difference (MCID). The MCIDs for the MBI and Fugl-Meyer assessments are 1.85 points [34] and 6.5 points [35], respectively. Therefore, we concluded that robot-assisted TOT incorporating HRCIFCT can be used as a safe and effective exercise protocol to improve the UL function of stroke patients. Some points require further discussion here relating the sites and time lapse following stroke onset among different patients. When interpreting these results, it is important to note that the five patients whom we selected were all in the early subacute phase of stroke (7 days to 3 months) [35]. Therefore, our interpretation only applies to stroke patients in this phase.

The Fugl-Meyer SEC score of subject 5 increased by 14 points after the final treatment. A possible reason for this increase could be the onset time of this patient (9 days). Studies have shown that early rehabilitative interventions are more effective than late interventions for restoring patients' functions and reducing the degree of disability. Despite the existing cognitive impairment of subject 1 (MMSE 17), this individual's posttraining Fugl-Meyer SEC score increased by 12 points. There are two possible reasons for this result. First, the duration of onset (16 days) for this patient was short, and second, the TOT centered on virtual reality games, which may be beneficial for improving cognitive function, as the results of a previous study also indicate [36].

Of the five patients, subject 4 showed the least improvement for their MBI score (10 points). Possible reasons for this result include the longer lapse between the stroke and treatment onset and a higher basal value, which resulted in just a small change in the patient's MBI score. Subject 2 showed the greatest improvement in their MBI score, amounting to 30 points after 15 sessions. A possible reason for this result is insignificant injury at the sites of the cerebral infarction in the brain stem and in the cortical spinal tract (CST), which is the key conduction tract that affects motor function. Significant damage to the CST can affect the performance and recovery of motor function [37]. Both the MBI and the Fugl-Meyer scores of subject 1 revealed progress after the training despite the patient's cognitive impairment. Therefore, we believe that patients with cognitive impairment will also have good therapeutic outcomes after receiving appropriate rehabilitative therapy.

## 5. Conclusion

The results of our experiments confirmed that robot-assisted TOT incorporating HRCIFCT can facilitate the recovery of stroke patients' UL function, even when cognitive dysfunction exists. Our findings also demonstrated that this robot-assisted rehabilitation system, entailing the application of HRCIFCT, is safe and effective. However, we conducted a small observational study; further research is required to confirm these results.

## Figures and Tables

**Table 1:** Profiles of the stroke patients.

Patient code	Age (years)	Sex	Type of stroke	Days since stroke	Impaired limb	MMSE	Fugl-Meyer SEC	MBI
S1	66	Female	CI	16	Left	17	25	68
S2	63	Male	CI	65	Right	30	19	55
S3	56	Female	CI	61	Left	27	27	71
S4	75	Male	CI	71	Right	27	27	71
S5	68	Male	CI	62	Right	30	20	47

CI = cerebral infarction; MMSE = Mini-Mental State Examination; Fugl-Meyer (SEC) = Fugl-Meyer assessment for shoulder–elbow, coordination; MBI = modified Barthel Index.

## Data Availability

The data that support the findings of the study are available from the corresponding author on reasonable request.
